# Breakfast consumption among Saudi primary-school children relative to sex and socio-demographic factors

**DOI:** 10.1186/s12889-020-8418-1

**Published:** 2020-04-06

**Authors:** Hazzaa M. Al-Hazzaa, Abdulrahman M. Alhowikan, Maha H. Alhussain, Omar A. Obeid

**Affiliations:** 1 Lifestyle and Health Research Center, Health Science Research Center, Princess Nourah bint Abdulrahman University, P. O. Box: 93216, Riyadh, 11673 Saudi Arabia; 2 Department of Physiology, Faculty of Medicine, King Saud University, Riyadh, Saudi Arabia; 3 Department of Food Science & Nutrition, College of Food & Agricultural Sciences, King Saud University, Riyadh, Saudi Arabia; 4 Department of Human Nutrition and Food Sciences, American University of Beirut, Beirut, Lebanon

**Keywords:** Breakfast, Children, Gender, Skipping breakfast, Saudi Arabia, Socio-demographic factors

## Abstract

**Background:**

Breakfast is an important meal that provides essential nutrients and energy. However, few comprehensive studies have reported breakfast habits and related behaviors among Saudi children. This study investigated breakfast consumption patterns and the associations of socio-demographic variables with daily breakfast intake among Saudi children.

**Methods:**

A multistage stratified cluster random sampling technique was used to select 1051 elementary school boys and girls in Riyadh. Body weight and height were measured and body mass index (BMI) was computed. The breakfast eating habits and behaviors were assessed using a specifically designed self-reported questionnaire that was completed by the children’s parents.

**Results:**

More than 79% of children skipped daily breakfast, with no significant sex difference. Children in private schools consumed breakfast more frequently than those attending public schools. Multivariate analyses showed that boys in private schools had a significantly higher intake of breakfast than that in boys in public schools, yet, boys in public schools had significantly higher BMI than boys in private schools. Using logistic regression while adjusting for confounders showed insignificant effect for parent education. Among breakfast eaters, spread cheese sandwiches were consumed most frequently, followed by fried egg sandwiches and breakfast cereals. Full-fat milk, tea with milk, water, and fruit juice were the most consumed drinks. Girls consumed significantly more fresh fruits during breakfast than did boys. Mothers prepared breakfast at home most of the time (84.5%). Parents appeared mostly satisfied with the breakfast consumed by their child at home and placed high importance on breakfast compared to lunch or dinner.

**Conclusions:**

The proportion of school children who ate daily breakfast at home was low, which may have implications for children’s school performance. Effort is needed to promote daily breakfast consumption among Saudi school children and to introduce appropriate interventions aimed at promoting daily breakfast consumption among Saudi children.

## Background

Breakfast is an important meal of the day, providing essential nutrients to support normal growth and re-fueling energy after long hours of sleep [1]. In addition, breakfast consumption can improve cognitive learning and academic performance [2–6]. In contrast, skipping breakfast in adolescents is associated with less healthy behaviors such as lower physical activity levels [7] and poorer food choices [1]. Moreover, family environment and structure have been shown to influence breakfast behaviors. Children living with both parents were more likely to consume breakfast compared to those living with a single parent [8, 9]. Furthermore, a study with a large number of adolescents from ten European cities reported that the factors most associated with breakfast consumption included girls with highly educated mothers, boys with traditional families, boys encouraged by their parents, and girls whose peers ate healthy diets [10]. Barriers to breakfast consumption are diverse; however, among elementary school children in Bangkok, the key barriers were lack of time, waking late, and no breakfast prepared at home [11].

Skipping breakfast predisposes children to obesity. In the United States, adolescents who consumed breakfast more often had a lower body mass index (BMI) than that in those who skipped breakfast [12]. It has been estimated that skipping daily breakfast among Canadian pre-school children nearly doubled their chances of being overweight [13]. Childhood obesity was also associated with those who, over the long-term, skipped breakfast and who consumed bread and sugar-sweetened drinks rather than fruits and vegetables [14, 15]. Moreover, a recent meta-analysis involving 106,935 participants revealed that skipping breakfast was associated with a significantly increased risk of developing type 2 diabetes mellitus [16]. In addition, skipping breakfast affects the adequacy of nutrient intake. A multicenter European study involving a large number of adolescents aged 12· 5-17·5 years from ten cities found lower vitamin D and vitamin C concentrations in boys and girls who skipped breakfast compared to those who ate breakfast [17].

Not only having breakfast but also the type of breakfast may be of significant importance in meeting nutritional needs. A large body of epidemiological research has consistently reported that higher consumption of ready-to-eat (RTE) cereals by children is more likely to meet their recommended nutrient intakes [18–20]. Breakfast cereal consumption was associated with higher dietary vitamins and mineral intake and healthy eating index and was not related to increased total energy or sodium intakes [21]. However, breakfast consumption of RTE cereals may contain low or high amounts of sugar with the latter case of nutritional concern. A randomized experiment revealed that high-sugar cereals increased children’s total sugar consumption and reduced the overall nutritional quality of their breakfast when compared to serving low-sugar cereals and that children were more likely to consume low-sugar cereals when provided with fruits, which is considered a healthier breakfast choice [22].

Although breakfast is well-recognized for its benefits for the well-being of children, insufficient studies have been conducted on breakfast habits and related behaviors among Saudi school children and adolescents [23–26]. Earlier breakfast findings in Saudi children revealed a low percentage of breakfast skippers (23, 24); however, these studies were conducted two decades ago. Furthermore, a large epidemiological study involving Saudi adolescents from three urban cities (Riyadh, Jeddah, and Al-Khobar) found that only 28.7% of boys and 20.6% of girls had daily breakfast intake [25]. Indeed, the proportion of breakfast skippers among Saudi adolescents appears much larger than the estimated 10-30% reported worldwide [27]. Factors associated with breakfast skipping among Saudi adolescents include higher BMI and waist to height ratio [28] and inadequate sleep duration [29]. The differences between boys and girls in food and beverage choices for breakfast meals were investigated among young Saudis aged 12–18 years in the Al Qassim region [26]. The findings indicated that boys had healthier choices and skipped breakfast less often than did girls. Additionally, girls in schools ate sandwiches, pizza, chips, and cookies for breakfast more often than boys did, whereas boys more frequently drank milk and juice in school than did girls [26].

It is evident from the above-mentioned studies that breakfast consumption is an important and healthy dietary habit and that skipping breakfast is an unhealthy eating lifestyle with negative influences on child health, well-being, and academic and cognitive performance. However, although we have some knowledge about Saudi adolescent’s breakfast habits, data on breakfast habits and associated behaviors in young children are insufficient, despite recent massive lifestyle and nutritional transitions among Saudi children and adolescents. Therefore, the purpose of the present study was to investigate the breakfast consumption patterns, including foods and beverage choices and preferences, and to examine the associations of socio-demographic variables and BMI with daily breakfast intake among Saudi children attending elementary schools in Riyadh.

## Methods

### Sample size and sampling technique

This cross-sectional study was conducted in Riyadh city from March to May 2017. Detailed sampling information have been published elsewhere [30]. Briefly, the study included Saudi children enrolled in primary schools (grades 1–6), who did not suffer from any medical condition that was related to eating disorders or allergy to foods. Participants’ selection was based on a multistage stratified cluster random sampling technique. Stratification was based on geographical areas (east, west, north, and south) and boys’ and girls’ schools. Within each geographical area, schools were randomly selected and classes were chosen randomly from each of the six grades. Invitation to participate in the study was then given to every student in the chosen classes. The sample size was computed with a population proportion equal 0.50, with a confidence level of 95% and a margin of error of 5%. Further, to account for any missing data, non-responders, and the clustered design effect, additional 20% of participants were added to the computed sample size. Thus, the final sample size become 920 boys and girls.

There are normally about 25 Saudi students in each class in public schools and nearly 15 Saudi students in each class in private schools. Data were collected from 12 elementary schools in the city of Riyadh with 72 classes, randomly located in the four geographical areas of the city (east: 2 schools [12 classes] from each of boys’ and girls’ schools; west: one [6 classes] boy’s school and one [6 classes] girl’s school; north: 2 schools [12 classes] from each of boy’s and girl’s schools; south: one [6 classes] boy’s school and one [6 classes] girl’s school). Trained research assistants collected the data with supervision from two of the main investigators. After obtaining consent forms from parents, weight and height were measured at schools by the research assistants and the returned questionnaires from parents were collected from the students in school.

### Ethical considerations and institutional review board (IRB) approval

The IRB of King Saud University approved the study (17/0064/IRB). The research procedures were consistent with the principles expressed in the Declaration of Helsinki. Data confidentiality was ensured by coding and storage in restricted access files. The children and their parents were free to withdraw from the study at any time if they expressed unwillingness to answer the questionnaire. Approval to conduct this study in primary schools was obtained from the directorate of schools at the Ministry of Education and the principals of the selected schools. In addition, written informed consent was obtained from all parents.

### Anthropometric measurements

Anthropometric measurements included body weight and height of the children as well as the calculated body mass index (BMI). Measurements were performed in the morning by a trained researcher according to written standardized procedures. Body weight was measured to the nearest 100 g using calibrated portable medical scales (Seca, Germany). All measurements were conducted with minimal clothing and without shoes. Height was measured to the nearest centimeter using a calibrated measuring rod while the subject was in a full standing position without shoes. BMI was calculated as the ratio of weight in kilograms divided by the squared height in meters.

### Breakfast eating habits and behaviors

The breakfast eating habits, food choices, and behaviors were assessed using a specifically designed self-reported questionnaire that was completed by the children’s parents (see supplementary file entitled Additional file 1). In addition, the questionnaire included information on father’s and mother’s ages, father’s and mother’s educational levels, family income, and type of housing. Moreover, parents were asked who prepared breakfast for the child, how satisfied they were with the breakfast consumed by the child, how important they felt the breakfast meal was for their child, and if they believed the breakfast meal was important for their child’s academic performance. Other questions were related to breakfast choices and behaviors. Breakfast food choice was defined as what the child chose to eat to break their fast at home or on their way to school or at school from early morning until 10 am during school days and from early morning until 11 am on weekends. A variety of common breakfast choices of were provided in the questionnaire including egg, cheese, peanut butter, or hummus sandwiches; pizza; ready-to-eat cereals; potato; sausage; cookies; or muffins. Parents were also asked to answer questions based on the child’s usual dietary habits in a typical week. The questionnaire was reviewed and agreed upon by three experts in the field of nutrition and dietary habits.

### Data and statistical analysis

Data were entered into a data file, checked, cleaned, and analyzed using IBM SPSS Statistics for Windows, version 22.0. Descriptive statistics were reported as means and standard deviations. Categorical variables were reported as proportions and Chi-square tests. Differences between boys and girls in anthropometric measurements were tested using t-tests for independent samples. The differences in breakfast intake per week relative to sex and school type were compared by two-way analysis of covariance (ANCOVA) while controlling for age. Logistic regression analysis was also used to test the associations of sociodemographic variables with daily versus non-daily breakfast intake. Finally, we used two-way multivariate analysis of covariance (MANCOVA) to examine differences in average breakfast intake and BMI as stratified by sex and school type while controlling for several sociodemographic factors (child’s age, father age, mother age, father education, mother education, family income, and housing size) and we included BMI as an outcome to check if the interaction effect between sex and school type was independent of BMI differences between boys and girls in public versus private schools. The Bonferroni test was used to test for between-subject effects. The alpha level was set at 0.05 and *p*-values less than the alpha level were considered significant.

## Results

The final number of participants was 1051 children (523 boys and 528 girls) between the ages of 5.9 and 13.4 years, with a mean age of 9.2 years, which was similar between boys and girls. The response rate by the parents was 94.5%. Students coming from public schools represented two-thirds of the participating sample. Parents answering the questionnaire appeared to have no problems with the phrases used in the questionnaire, as evident by their high response rate and lack of comments regarding the clarity of the questions on the returned questionnaires.

Analysis of the demographic characteristics of the participants (not shown in tables) indicated significant (*p* <  0.001) differences in parent’s responses between boys’ and girls’ schools. Fathers answered more boy school’s questionnaires than did mothers (51.3% versus 33.5%) whereas mothers answered more girl school’s questionnaires than did fathers (59.5% versus 43.8). On average, fathers were older than mothers and a higher percentage of them had post-graduate degrees. However, mothers had a higher percentage of college degrees than that of fathers. In addition, approximately 50% of the parents’ incomes ranged between 5001 and 15,000 Saudi Riyals (1334–4000 US$).

Table 1 presents the descriptive characteristics of the participants, average breakfast intake per week, and the proportion of children with daily breakfast intake, stratified by sex and school type. Boys were significantly (*p* <  0.001) heavier and had a significantly (*p* <  0.001) higher BMI. The average breakfast consumption by the children was 3.51 days per week with no significant difference between boys and girls. However, there was a significant (*p* = 0.001) sex by school interaction in average breakfast intake. The proportion of daily breakfast intake was almost 21%, with no significant difference in the proportions of boys and girls consuming daily breakfast (19.3% versus 22.1%). However, a significantly higher proportion of girls in public schools (21.4%) had daily breakfast intake compared to that in boys (13.3%) (*p* = 0.005), whereas a significantly higher proportion of boys in private schools ate daily breakfast compared to boys in public schools (32.5% versus 13.3%, *p* <  0.001). Moreover, two-way ANOVA (not shown in Table 1) revealed no significant difference in breakfast intakes (days per week) between boys and girls relative to age categories or child’s living status (living with both parents, with father, with mother, or with a relative). The results also showed no significant differences between boys and girls in breakfast intakes during weekdays (*p* = 0.601) or weekends (*p* = 0.154). However, there was more adherence to daily breakfast intake on weekends (41.3%) than on weekdays (28.9%).

**Table 1:** Descriptive characteristics of the participating children

Variable	Boys (*N* = 523)	Girls (*N* = 528)	All (*N* = 1051)	***p***-value ^**a**^
**Age (years)**	9.3 ± 1.7 (9.16–9.45)	9.2 ± 1.7 (9.06–9.35)	9.2 ± 1.7 (9.10–9.30)	0.165
**Body weight (kg)**	38.6 ± 23.2 (36.6–40.6)	33.2 ± 12.9 (32.9–34.3)	35.8 ± 18.9 (34.7–36.9)	**< 0.001**
**Body height (cm)**	133.6 ± 11.7 (132.6–134.6)	133.1 ± 12.5 (132.0–134.2)	133.4 ± 12.1 (132.7–134.1)	0.517
**Body mass index (BMI)**	20.8 ± 9.6 (20.0–21.6)	18.1 ± 4.4 (17.7–18.5)	19.4 ± 7.6 (18.9–19.9)	**< 0.001**
**Average breakfast intake (day/week)**	3.44 ± 2.3 (3.24–3.64)	3.58 ± 2.3 (3.38–3.78)	3.51 ± 2.3 (3.37–3.65)	0.350
Public school	3.07 ± 2.2 (2.88–3.26)	3.52 ± 2.3 (3.32–3.72)	3.30 ± 2.3 (3.16–3.44)	**Gender by school interaction = 0.001**
Private school	4.23 ± 2.3 (4.03–4.43)	3.68 ± 2.3 (3.48–3.87)	3.95 ± 2.3 (3.81–4.09)	
**Daily breakfast intake (%)**	19.3	22.1	20.7	0.285
Public school	13.3	21.4	17.3	**0.005**
Private school	32.5	23.6	27.9	0.070
*p*-value	**< 0.001**	0.736	**< 0.001**	

Data are means ± standard deviations (95% CI) or percentage

^a^T-test for independent samples (anthropometrics), 2-way ANCOVA (with average breakfast intake (day/week) as adjusted for age) or Chi Squares tests for the proportions (daily breakfast intake (%))

The types of breakfast choices (%) consumed at home by Saudi children relative to sex are shown in Table 2. A variety of foods were consumed during breakfast by the Saudi children. Spread cheese sandwiches (42.2%) were consumed most frequently, followed by fried egg sandwiches (41.1%), breakfast cereals (34.3%), and boiled egg sandwiches (27.1%). Among the kind of cereals consumed at breakfast, cornflake cereals were predominant (23.6%). Cereals such as bran with honey and nuts or oat cereals were consumed least frequently. Less healthy foods such as donuts, hot dogs, hamburger, cookies, biscuits, and French fries were minimally consumed (2.4–4.5%). In general, the majority of children seemed to prefer their breakfast in the form of sandwiches. There were significant differences in eating patterns between boys and girls. There were also significant differences between boys and girls in their preferences for breakfast. Some popular Mediterranean breakfast choices like fava beans (*Foul*), Labneh sandwich, thyme sandwich, and falafel sandwich were fairly consumed by children as breakfast. Additionally, traditional foods such as sweet Arabic pastries, dates, or Marase’a (home-made wheat bread) was were not often eaten by children at breakfast time. Furthermore, children in public school consumed significantly more spread cheese sandwiches than those in private schools (44.7 versus 37.1), whereas children in private schools seem to prefer to breakfast cereals (40.9% versus 31.2%), Nutella sandwich (24.4% versus 17.9%), Falafel sandwich (fava beans) (8.8% versus 5.8%), and pancakes (7.9% versus 4.9%) compared to children in public schools.

**Table 2:** Types of breakfast (%) usually consumed by Saudi children relative to gender (more than one choice was possible)

Variable	Boys	Girls	All	***p***-value ^**a**^
Spread cheese sandwich	39.2	45.3	42.2	**0.046**
Fried egg sandwich	45.1	37.1	41.1	**0.008**
Breakfast cereals	30.2	38.4	34.3	**0.003**
Boiled egg sandwich	27.5	26.7	27.1	0.762
Nutella sandwich	16.6	23.5	20.1	**0.006**
Tuna sandwich	13.6	20.1	16.8	**0.005**
Fava beans (Foul)	17.0	14.4	15.7	0.242
Labneh sandwich ^b^	10.5	17.8	14.2	**0.001**
Croissant	9.9	11.6	10.8	0.399
Cheese pie (*Fataer Jubin*)	9.8	10.4	10.1	0.367
Thyme sandwich	6.9	11.2	9.0	**0.015**
Peanut butter sandwich	8.0	10.0	9.0	0.256
Falafel sandwich	7.5	6.1	6.8	0.367
Jam sandwich	7.1	5.1	6.1	0.184
Pancake	4.6	7.2	5.9	0.073
Solid cheese sandwich	5.0	6.6	5.8	0.251
Labneh pie (*Fataer Labneh*)	4.0	6.4	5.2	0.078
Thyme pie (*Fataer Zatar*)	3.8	6.4	5.1	0.055
Mortadella sandwich	4.0	5.2	4.7	0.322
Yogurt-with or without fruits	2.9	6.3	4.6	**0.009**
Oreo biscuit/other types of biscuit	4.4	4.5	4.5	0.908
Donuts	2.7	4.4	3.5	0.140
Pizza	3.3	3.6	3.4	0.756
Hot dog	3.6	3.0	3.3	0.586
Hamburger	3.4	2.1	2.8	0.179
Cake or cookies	2.7	2.8	2.8	0.871
Chickpeas (Hummus) sandwich	2.7	2.3	2.5	0.673
French fries	2.5	2.3	2.4	0.821
Baked potatoes	0.0	1.5	0.8	**0.005**
Other kinds of breakfast	2.9	5.1	4.0	0.063

^**a**^Chi Squares tests of the proportions for significant difference between boys and girls

^b^Soft, cream cheese made from strained yogurt, popular in Middle Eastern cuisine

Table 3 shows breakfast intake preferences at home among Saudi children relative to sex. Most (71.5%) boys and girls preferred to consume breakfast cereals with sugar, while a much smaller proportion (18.0%) preferred cereals with fruits, without any significant difference between boys and girls. In addition, a higher percentage of girls (49.6%) than boys (43.2%) consumed fresh fruit at breakfast. Apple consumption (21.8%) came first, followed by banana (21.2%), orange (13.5%), and grapes (11.0%), with girls consuming more apples and grapes compared to that in boys. Furthermore, most children (97.1%) consumed drinks during breakfast time. Full-fat milk (40.8%), tea with milk (34.5%), water (29.9%), and fruit juice (24.4%) were the drinks most consumed by the children. Girls showed significantly (*p* = 0.014) higher water intake at breakfast than that in boys.

**Table 3:** Breakfast intake preferences by Saudi children relative to gender (more than one choice was possible)

Variable	Boys	Girls	All	***p***-value ^**a**^
**Preference for cereal with/without added sugar (%)**				0.374
*With added sugar*	72.9	70.1	71.5	
*Without added sugar*	27.1	29.9	28.5	
**Preference for cereal with or without fruit (%)**				0.439
*With fruit*	16.8	19.1	18.0	
*Without fruit*	83.2	80.9	82	
**Percentage of fresh fruit eaten with breakfast (%)**	43.2	49.6	46.4	**0.037**
**Types of fruits most consumed with breakfast (%):**				
*Apple*	18.0	25.6	21.8	**0.003**
*Banana*	19.1	23.3	21.2	0.098
*Orange*	12.8	14.2	13.5	0.509
*Grape*	9.0	13.1	11.0	**0.035**
*Pear*	3.1	5.7	4.4	**0.038**
*Strawberries*	2.5	5.7	4.1	**0.009**
**Percentage of drinks consumed with breakfast (%)**	98.1	96.0	97.1	**0.048**
**Types of drinks most consumed with breakfast (%):**	0.4	0.8	0.6	0.420
*Full fat milk*	42.3	39.4	40.8	0.345
*Tea with milk*	34.4	34.7	34.5	0.934
*Water*	26.4	33.3	29.9	**0.014**
*Fruit juice*	22.0	26.7	24.4	0.075
*Tea*	13.2	12.3	12.7	0.668
*Fruit drink*	7.3	8.3	7.8	0.519
*Low fat milk*	4.8	9.3	7.0	**0.004**
*Soft drink (soft beverage)*	5.9	1.7	3.8	**< 0.001**

^**a**^Chi Squares tests of the proportions for significant difference between boys and girls

Table 4 shows the timing of breakfast consumption and parent’s perception of their child’s daily or non-daily breakfast intake. There was no significant difference in daily breakfast frequency between children consuming breakfast before or after 6:00 am (schools start at 7:00 am). In addition, mothers and domestic helpers prepared breakfast 85 and 8.6% of the time, respectively. More than 40% of the parents were very satisfied with their children’ breakfast intake. Daily intake of breakfast was significantly higher among the children of parents who were very satisfied with their children’ breakfast (*p* = 0.004). In addition, parents placed high importance (91.2%) on the breakfast intake of their children. However, this was not significantly reflected in daily breakfast intake. Compared to lunch and dinner, 80.0% of the parents considered breakfast to be the most important meal of the day for their children. The children of parents with strong opinions on breakfast as a meal were more likely to have daily breakfast intake. Also, nearly 74% of the parents considered having enough protein in their children’s breakfast to be very important. Furthermore, 93% of parents believed that eating breakfast improved their child’s academic performance. However, this did not influence the breakfast intake of the children.

**Table 4:** Timing of breakfast consumption and parent’s perception of child’s breakfast intake

Variable (percent within item relative to total)	Breakfast Intake (%)		***p***-value *
	Daily	Non-daily	
**Time breakfast consumed by the child (%)**			0.311
Before 6:00 am (47.4%)	24.8	75.2	
After 6:00 am (52.6%)	23.1	76.9	
**Who prepare the breakfast for the child? (%)**			**0.039**
Mother (84.5%)	20.3	79.7	
Father (0.5%)	20.0	80.0	
Domestic helper (8.6%)	33.7	66.3	
Sister/brother (3.0%)	16.1	83.9	
The child himself/herself (2.1%)	13.6	86.4	
Brought ready from the market (0.3%)	0.0	100.0	
Nothing is prepared at home (0.9%)	0.0	100.0	
**How much Satisfied with the breakfast consumed by your child at home? (%)**			**0.004**
Satisfied (40.3%)	24.0	76.0	
Somewhat happy (47.3%)	21.5	78.5	
Not satisfied (12.4%)	10.3	89.7	
**As a meal, how important is your child’s breakfast for you? (%)**			0.389
Very important (91.2%)	21.4	78.6	
Somewhat important (8.0%)	15.7	84.3	
Not important (0.8%)	12.5	87.5	
**In your opinion, which is the most important meal of the day for your child? (%)**			**0.001**
Breakfast (80.2%)	23.2	76.8	
Lunch (17.7%)	12.0	88.0	
Dinner (2.1%)	4.5	95.5	
**How important having enough protein in your child’s breakfast? (%)**			0.395
Very important (73.7%)	21.3	78.7	
Not important (4.5%)	29.5	70.5	
I do not know (21.8%)	20.4	79.6	
**Do you think (as a parent) that having breakfast improves child’s academic performance? (%)**			0.141
Yes (93.0%)	21.4	78.6	
No (0.2%)	50.0	50.5	
I do not know (6.8%)	12.9	87.1	

*Chi Squares tests of the proportions for significant difference between boys and girls

Additionally, there was a low percentage (nearly 1%) of lactose intolerance among the children, with more boys (1.6%) than girls (0.2%) having this nutritional disorder. The three most common reasons for the lack of regular breakfast at home were not feeling hungry (nearly 50%), waking late (21.2%), and no time to eat breakfast at home but provided money to buy food at school (17.1%). The main types of sandwiches eaten by children on their way to schools were egg (45.3%) and cheese (nearly 35%) sandwiches.

Table 5 presents the socio-demographic characteristics of the participants stratified according to daily or non-daily breakfast intakes (%). There were no significant differences in many variables between children consuming daily or non-daily breakfast. However, there were significant differences between daily versus non-daily breakfast consumers in father’s education (*p* <  0.001), mother’s education (*p* = 0.004), and family income (*p* = 0.001), with a clear positive trend between daily breakfast intake and increasing father education, mother education, and family income.

**Table 5:** Socio-demographic characteristics of the participants relative to daily or non-daily breakfast intake (mean and SD or percentage)

Variable (percent within item relative to total)	Breakfast Intake		***p***-value ^a^
	Daily	Non-daily	
**Age (years)**	9.1 ± 1.6	9.3 ± 1.8	0.266
**Body weight (kg)**	34.7 ± 18.7	36.2 ± 19.1	0.332
**Height (cm)**	132.4 ± 12.0	133.6 ± 12.2	0.188
**BMI**	19.1 ± 1.7	19.5 ± 1.7	0.441
**Average breakfast intake (day/week)**	7.0	2.7 ± 1.7	**< 0.001**
**Parent answering the questionnaire (%)**			0.596
Mother (53.5%)	21.5	78.5	
Father (43.1%)	19.6	80.4	
Someone else (3.4%)	25.7	74.3	
**The child is living with (%)**			0.692
Both parents (94.3%)	20.5	79.5	
Mother (4.2%)	22.7	77.3	
Father (1.3%)	30.8	69.2	
Relative (0.2%)	0.0	100.0	
**Number of family members living in the house (%)**			0.609
2–5 (32.6%)	19.5	80.5	
6-9 (60.8%)	22.0	78.0	
10 + (6.6%)	19.1	80.9	
**Father’s age (%)**			0.476
< 30 years (0.3%)	0.0	100.0	
30-39 years (27.2%)	23.7	76.3	
40-49 years (46.5%)	20.0	80.0	
50–59 years (22.5%)	20.4	79.6	
60 + years (3.5%)	13.9	86.1	
**Mother’s age (%)**			0.095
< 30 years (8.7%)	15.6	84.4	
30-39 years (54.1%)	22.5	77.5	
40-49 years (32.6%)	20.2	79.8	
50–59 years (4.3%)	13.3	86.7	
60 + years (0.3%)	66.7	33.3	
**Father’s education (%)**			**< 0.001**
Intermediate or less (11.8%)	12.3	87.7	
High school (32.0%)	14.8	85.2	
University degree (43.2%)	24.9	75.1	
Post graduate degree (12.9%)	30.8	69.2	
**Mother’s education (%)**			**0.004**
Intermediate or less (17.9%)	18.4	81.6	
High school (31.9%)	15.2	84.8	
University degree (46.9%)	25.2	74.8	
Post graduate degree (3.4%)	25.7	74.3	
**Family income (%)**^**b**^			**0.001**
5000 SR or less (13.4%)	13.6	86.4	
5001–10,000 SR (26.4%)	16.2	83.8	
10,001–15,000 SR (25.1%)	21.1	78.9	
15,001–20,000 SR (18.3%)	22.2	77.8	
20,001–25,000 SR (8.1%)	36.3	63.7	
25,001 + SR (8.8%)	25.3	74.7	
**Type of housing living in (%)**			0.295
Apartment (19.8%)	16.2	83.8	
One floor in a villa (28.2%)	22.3	77.7	
Small villa (less than 500 m^2^) (26.0%)	23.1	76.9	
Medium size villa (500–1000 m^2^) (21.9%)	19.5	80.5	
Large villa (more than 1000 m^2^) (4.1%)	26.2	73.8	

^a^Chi-Square tests of proportions

^**b**^*SR* Saudi Riyal = 3.75 US$

Table 6 displays the results of logistic regression analysis adjusted for age, weight, and BMI for selected sociodemographic variables relative to daily versus non-daily breakfast intake. Only age was a significant covariate, as well as mother age of less than 30 years and 50–59 years. Father’s education, mother’s education, and family income, which were significant influencing factors of daily breakfast intake in Table 5, were insignificant in the logistic regression analysis.

**Table 6:** Results of logistic regression analysis, adjusted for age, weight and BMI, for selected sociodemographic variables relative to daily versus non-daily breakfast intake among Saudi children

Variable	Daily versus non-daily breakfast intake ^a^			
	aOR	(95% CI)	SEE	***p-****value*
**Age**	0.953	0.824–1.102	.074	**0.008**
**Weight**	0.993	0.954–1.033	0.020	0.717
**BMI**	1.017	0.928–1.115	0.047	0.717
**Gender** (girls = ref)	1.00			
Boys	0.830	0.582–1.184	0.830	0.303
**School type** (private = ref)	1.00			
Public	0.874	0.582–1.300	0.203	0.506
**No. of Family member in the house** (≥ 10 = ref)	1.00			
6-9	0.716	0.352–1.456	0.362	0.356
2-5	0.604	0.274–1.329	0.403	0.210
**Father age** (60 + years = ref)	1.00			
50–59	1.756	0.477–6.460	0.655	0.397
40-49	1.570	0.418–5.889	0.675	0.504
30-39	2.230	0.565–8.804	0.701	0.252
**Mother age** (60 + years = ref)	1.00			
50–59	0.047	0.003–0.816	1.451	**0.036**
40-49	0.085	0.005–1.331	1.402	0.079
30-39	0.095	0.006–1.516	1.412	0.096
< 30	0.044	0.002–0.770	1.465	**0.033**
**Father education** (postgraduate = ref)	1.00			
College degree	0.871	0.520–1.465	0.264	0.608
High school	0.551	0.301–1.011	0.309	0.054
< High school	0.555	0.231–1.331	0.447	0.187
**Mother education** postgraduate = ref)	1.00			
College degree	1.853	0.665–5.167	0.523	0.238
High school	1.399	0.481–4.068	0.545	0.538
< High school	2.375	0.771–7.323	0.574	0.132
**Family income** (> 25,000 = ref)	1.00			
20,001 - 25,000	1.759	0.819–3.779	0.390	0.148
15,001 – 20,000	1.334	0.670–2.655	0.351	0.412
10,001 – 15,000	1.006	0.507–1.999	0.350	0.986
5001 -10,000	0.893	0.435–1.830	0.366	0.786
≤ 5000	0.798	0.332–1.917	0.447	0.614

^a^Non-daily breakfast intake was used as a reference category. *aOR* adjusted odds ratio, *CI* confidence interval, *ref*. reference category, *SEE* standard error

The results of two-way MANCOVA for average breakfast intake and BMI stratified by sex and school type while controlling for socio-demographic factors are shown in Table 7. The findings indicated that sex was significant (*p* <  0.001), whereas school type was not (*p* = 0.070). However, the sex by school type interaction effects for breakfast intake were significant (*p* <  0.001. Boys in private schools had higher breakfast intake than that in girls, whereas girls in public schools had higher breakfast consumption than that in boys; however, boys in public but not in private schools were significantly heavier than girls.

**Table 7:** Results of multivariate analyses of average breakfast intake and body mass index (BMI) stratified by gender and school type, while controlling for confounders (data are mean and SD)

Variable	School type	Boys	Girls	***p***-value for between subjects effects
Average breakfast intake (day/week)	Public	3.1 ± 2.2 (2.7–3.4)	3.5 ± 2.3 (3.2–3.9)	Gender: 0.414 School type: 0.053 Gender by school type interaction: **0.003**
	Private	4.3 ± 2.3 (3.6–5.0)	3.7 ± 2.3 (3.2–4.2)	
BMI	Public	21.8 ± 11.6 (20.1–23.5)	18.1 ± 4.5 (17.4–18.8)	Gender: **< 0.001** School type: 0.229 Gender by school type interaction: **0.001**
	Private	18.9 ± 4.8 (18.8–20.2)	18.2 ± 4.3 (17.2–19.2)	

*P*-values for multivariate tests: child age: < 0.001; father age: 0.881 mother age: 0.006; father education: 0.054; mother education: 0.537; family income: 0.041; housing size: 0.070; gender: < 0.001; school type: 0.070; Gender x school type: < 0.001

## Discussion

This comprehensive study investigated breakfast habits and related behaviors among Saudi children in primary schools in Riyadh using a sufficient and representative sample randomly chosen from both public and private schools within the large metropolitan city of Riyadh. The main findings of the present study indicated that more than 79% of the sampled children skipped daily breakfast with no significant sex differences. Multivariate analyses showed that boys in private schools had significantly higher intake of breakfast than that among boys in public schools; however, boys in public schools had significantly higher BMI than that in boys in private schools. Parents’ education and family income significantly influenced breakfast consumption. In addition, significant sex by school type interactions were observed.

Comparison of our findings on breakfast skipping with those of earlier local studies revealed many differences. Earlier studies conducted in Saudi school students reported a slightly lower percentage of breakfast skipping by Saudi children than that in the current study [23, 24]. Among Saudi students aged 5–18 years in Jeddah city, the prevalence of breakfast skipping was nearly 15%, and there were no differences relative to age, sex, BMI, or social class. The study also found that breakfast skipping was associated with poor school performance [23]. Besides having been conducted a long time ago, the main focus of the previous study was anemia in school students and used a self-reported questionnaire even in young students aged 9–12 years [23]. In another study conducted in Riyadh city, 16.5% of the school girls did not eat breakfast at home and relied on snacks from the school canteen, which consisted mostly of chocolate bars, biscuits, and potato chips along with soda drinks [24]. Additional differences between our findings regarding breakfast skipping and those of the earlier local studies may also reflect lifestyle changes of Saudi children and adolescents (including nutritional patterns) over the past three decades [25], which is the period between the current and previous studies.

The frequency of regular breakfast consumption by the Saudi children in the present study was lower than that reported in other countries. For an instance, a study involving 41 countries participating in the Health Behavior in School-aged Children (HBSC) study found that daily breakfast consumption among students aged 11–15 year varied from 33% in Greek girls to 75% among Portuguese boys [31]. Furthermore, only 30% of Sri Lankan children consumed breakfast at home [32]. However, different surveys can produce different findings. Contrary to the non-significant sex difference in the proportion of daily breakfast intake observed in the present study, female adolescents from neighboring Bahrain [33] skipped breakfast significantly more often than did male adolescents (62.8% versus 37.2%).

Previous studies have shown that numerous factors can influence breakfast intake, including sex, family structure, and socio-economic status [2, 7, 9, 34, 35]. Our findings in the present study indicated that there was no significant difference in daily breakfast intake relative to the living status of the child (living with father, mother, or both parents), the number of family members living in the home, or father’s or mother’s ages. However, research elsewhere revealed that children living with both parents had higher chances of breakfast consumption compared to those living with a single parent [8, 9]. In addition, a decrease in daily breakfast consumption has been observed in children living in single-parent families [9] and an increase in breakfast intake was shown among children of two-parent families [35]. The difference between our findings and those from other studies may be due to the modest number of single mothers (4.3%) or fathers (1.3%) in the present study.

In the HBSC study, lower daily breakfast intake was associated with female sex, older adolescents, and living in single-parent families [31]. In the current study, there was no significant difference in the proportions of children consuming daily breakfast relative to sex. However, students in private schools had higher mean breakfast intakes. Children attending private schools had significantly higher parent education and family income levels, which may suggest that highly educated and affluent parents were more likely to have their children consume breakfast at home. In comparison, data from the National Health and Nutrition Examination Survey of the United States demonstrated that boys consumed daily breakfast more often than girls did [7]. Furthermore, a study with a large number of adolescents from ten European cities reported that the factors most associated with breakfast consumption included girls with highly educated mothers, boys with traditional families, boys encouraged by their parents, and girls whose peers ate healthy diets [10]. In the present study, logistic regression adjusted for confounders showed an insignificant effect of parent education. Nevertheless, parental education was found to influence breakfast intake in European children [34]. Moreover, family income was generally reported to be associated with breakfast consumption in children and adolescents [2]. However, among a group of school children from Riyadh surveyed two decades ago, there was no association between parents’ education and the frequency of breakfast intake at home [24]. Information on demographic and socioeconomic correlates of breakfast intake among children and youth is important for identifying children requiring intervention [36].

In the present study, the most common reasons for Saudi children to not have regular breakfast at home were not feeling hungry and waking up late or having no time to eat breakfast. This could due to the early start of Saudi schools (7.00 am), leaving little time in the morning before going to school for students who wake late. Most of the sampled children did not get sufficient sleep, as our current study showed that nearly 71% received less than the minimally recommended sleep duration of 9 h per night [37]. Similar findings were found among elementary school children in Bangkok, in which the key barriers to breakfast consumption were lack of time, waking late, and lack of breakfast prepared at home [11].

The present study did not find significant differences between daily versus non-daily breakfast consumption relative to body weight or BMI. However, obesity has previously been shown to be associated with skipping breakfast in Saudi adolescents [25, 28]. Elsewhere, daily breakfast intake appeared to offer control over body weight, improve blood lipids profile, and increase levels of physical activity among children aged 2–10 years from eight European countries [38]. Skipping breakfast is also associated with obesity in children and adolescents [13–15] and with increased risk of type 2 diabetes mellitus [16]. Furthermore, skipping breakfast among children and adolescents is related to increased cardio-metabolic risks, including increased obesity and elevated triglyceride, low-density lipoprotein cholesterol, and low-high density lipoprotein cholesterol levels compared to those in regular breakfast consumers [39]. In addition, numerous recent studies in adults have showed the adverse effects of skipping breakfast on cardiovascular (CV) and metabolic health, including increased risk of type 2 diabetes mellitus independent of lifestyle and baseline levels [40] and metabolic inflexibility as a result of prolonged fasting [41], as well as an association with higher risk of non-coronary and generalized atherosclerosis independent of the presence of conventional CV risk factors [42]. A review paper concluded that, despite the variability of the quality of breakfast within the reviewed studies, children who reported eating breakfast regularly were more likely to have superior nutritional profiles than those in peers who skipped breakfast [2]. Moreover, a multicenter European study involving 1058 adolescents aged 12.5–17.5 years from ten cities observed lower vitamin D and vitamin C concentrations in male and female breakfast-skippers than those in breakfast consumers [17].

A variety of foods were consumed by the Saudi children in the present study. However, spread cheese sandwiches, followed by fried egg sandwiches and breakfast cereals, were consumed most often. In addition, there were significant differences between boys and girls in breakfast intake preferences. Food choices made by children can be influenced by a complex range of variables. Such factors include family, friends, habit, socioeconomic status, health considerations, food availability, food appeal, food taste, ethical concerns, and wider societal trends. Epidemiological studies have consistently reported that a higher intake of RTE cereals by children is more likely to meet their recommended nutrient intakes [18, 19]. Furthermore, nearly 50% of the Spanish population aged 2–24 years eat RTE cereals and their macronutrient profile improved with increasing cereal consumption [20]. A recent systematic review on the benefits of RTE cereals concluded that breakfast cereal consumption was related to higher dietary vitamin and mineral intake and higher healthy eating index and was not linked to increased total energy or sodium intakes [21]. However, breakfast consumption of RTE cereals with high amounts of sugar presents nutritional concerns. In the current study, over 70% of Saudi children who ate breakfast cereals consumed products with sugar, while less than one-fifth of sample preferred cereals with fruits, without any significant sex differences. This may indicate that not many Saudi children ate healthy breakfast cereals rich in fiber and low in sugar. Furthermore, pizza, hot dogs, and hamburgers were reported as a breakfast choices by less than 10% of the Saudi children. In comparison, fast foods as a breakfast meal were the most preferred food by Siri Lankan children, followed by wheat flour-based breakfast items [32]. In the present study, Mediterranean food choices like fava beans (*Foul*) and thyme and falafel sandwiches were fairly consumed during breakfast. Such vegetable-based foods are considered healthy and have high levels of protein, fibers, vitamins, and minerals.

Our findings indicated that most children consumed drinks during breakfast time, with a significantly higher percentage of boys consuming drinks compared to that in girls. Full-fat milk, tea with milk, water, and fruit juice were the drinks most consumed by the children. Girls showed a significantly higher water intake with breakfast than that in boys. A previously published local study found that tea drinking was common among schoolgirls in Riyadh [24]. Moreover, a study involving adolescents aged 12–18 years from the Al Qassim region in central Saudi Arabia, showed contrasting findings in fluid intake between boys and girls during breakfast, with boys more frequently drinking milk and juice compared to girls, while the girls consumed more water compared to boys [26]. The current study also showed that a significantly higher percentage of girls consumed fresh fruits with breakfast, especially apples, bananas, oranges, and grapes, compared to that in boys, in contrast to earlier reports involving Saudi school children in Riyadh showing infrequent consumption of fresh fruits and vegetable by girls [24]. This difference may reflect changes over the two decades due to economic growth of the country and purchasing power of the families.

The results of the present study showed that Saudi parents placed very high importance on the breakfast meal of their children and that, compared to lunch and dinner, most parents considered their children’s breakfast to be the most important meal of the day. This finding agrees with the results of a study conducted in Siri Lanka, which reported that the mothers of Siri Lankan children considered breakfast to be an essential meal [32]. Furthermore, our results showed a significant trend toward higher daily breakfast intake when parents were satisfied with their children’s breakfast and that most parents believed that having breakfast can improve the academic performance of children. This finding agrees with previous research showing that breakfast consumption can improve cognitive learning and academic performance in children [2–6]. Thus, parental knowledge and emphasis on the importance of breakfast consumption were not lacking among most of the Saudi parents in the present study. However, a previous report recommended working with children, parents, and schools to generate positive knowledge and beliefs about breakfast consumption that might be beneficial in increasing the rate of breakfast intake among children [43].

Finally, this study has its strengths and limitations. The strengths include the large and representative sample of Saudi children from both public and private schools. However, the limitations of the current study include its cross-sectional design, which precludes us from determining a causal relationship between breakfast intake and respective variables. In addition, breakfast consumption and behaviors were assessed using a questionnaire completed by the child’s parent. Questionnaires are generally subject to recall bias and social desirability effects. Finally, although Riyadh is a cosmopolitan city with people from all parts of the country, the breakfast choices and preferences of the children in the present study may not exactly reflect those of children in other parts of the country, especially in rural areas.

## Conclusions

The present study revealed that about one-fifth of the sampled children consumed breakfast daily, with no significant difference between boys and girls. Children in private schools, however, consumed daily breakfast significantly more frequently than those children in public schools. Multivariate analyses showed that boys in private schools had a significantly higher intake of breakfast than that in boys in public schools, yet boys in public schools had a significantly higher BMI than that in boys in private schools. Using logistic regression while adjusting for confounders showed an insignificant effect of parent education. Spread cheese sandwich followed by fried egg sandwich and breakfast cereals were consumed most often by Saudi school children. Further, full-fat milk, tea with milk, water, and fruit juice were the drinks most consumed by the children. Girls consumed significantly more fresh fruits at breakfast than did boys. In addition, mothers generally prepared breakfast at home. There was a significant trend toward higher daily breakfast intake when parents were satisfied with their children’s breakfast. Parents also placed very high importance on breakfast compared to lunch or dinner. Overall, breakfast is a healthy and important dietary habit that is worth maintaining by school children. However, the proportion of Saudi children who skipped daily breakfast at home was high, which may have implications for their academic performance. Therefore, a concerted effort is needed to promote daily breakfast consumption among Saudi school children. Future studies may need to address the psychological, cultural, and environmental correlates associated with breakfast consumption among Saudi children. The introduction of appropriate interventions is also recommended to promote daily breakfast consumption among Saudi children.

## Supplementary information

**Additional file 1.** Breakfast eating habits and behaviors questionnaire. The questionnaire was developed by the authors and included items related to breakfast eating habits, food choices, and behaviors. The questionnaire also included information on demographic and socioeconomic status.

## Data Availability

All data generated or analyzed during this study are included in this published article. Any additional data are available from the corresponding author on reasonable request.

## Abbreviations

ANCOVA: Analysis of covariance
BMI: Body mass index
CV: Cardiovascular
HBSC: Health behavior in school-aged children
MANCOVA: Multivariate analysis of covariance
RTE: Ready-to-eat (RTE) cereals
