# Supplementary material for: Breakfast consumption among Saudi primary-school children relative to sex and socio-demographic factors
Source: BMC Public Health. 2020 Apr 6;20:448. doi: 10.1186/s12889-020-8418-1 (PMC7132954; doi:10.1186/s12889-020-8418-1)
Supplement: Supplementary file 1 — Additional file 1. Breakfast eating habits and behaviors questionnaire. The questionnaire was developed by the authors and included items related to breakfast eating habits, food choices, and behaviors. The questionnaire also included information on demographic and socioeconomic status. [file 12889_2020_8418_MOESM1_ESM.pdf]

SN	………….……

Breakfast Eating Habits and Behaviors questionnaire for Children 6-12 years old

(*To be filled by parents or the child's guardian*)

Dear parent or child's guardian: This questionnaire aims to collect information regarding your child's breakfast habits and associated behaviors. Breakfast meal is defined as the first consumed meal from the time of waking up and until **11** a.m. This is part of a large study that is conducted on elementary students in Riyadh. Kindly answer all the questions below by choosing the most precise and relevant answer. Note that the information you are disclosing will remain confidential and will only be used for scientific research.

Date of filling the questionnaire**:** ....

1-Who is the guardian filled the questionnaire: ☐ Mother ☐ Father ☐ Someone else: …………….

2- Sex of the child: ☐emale Male ☐

3- Age of the child (years): ……….

Please choose the most relevant answer by checking the corresponding box:

Part one:

4- With whom does the child lives?

☐ with both parents ☐ with the mother only ☐ with the father only

☐ with others (grandparents, etc.)

5- How many members of the family are living together in the house (please write the number in the box)? ☐

6- How many family members are below 18 years old (please write the number in the box)? ☐

7- How old is the father?

☐ Less than 30 ☐ 30-39 ☐ 40-49 ☐ 50-59 ☐ 60 or above

8- How old is the mother?

☐ Less than 30 ☐ 30-39 ☐ 40-49 ☐ 50-59 ☐ 60 or above

9-What is the father's highest education degree?

☐ Middle school or less ☐ High school ☐ College degree ☐ Masters or PhD

10-What is the father's highest education degree?

☐ Middle school or less ☐ High school ☐ College degree ☐ Masters or PhD

11- What is the average monthly income of the family?

☐ 5000 Riyal or less ☐ 5001-10000 Riyal ☐ 10001-15000 Riyal

☐ 150000-20000 Riyal ☐ 20001-25000 Riyal ☐ More than 25000 Riyal

12- What is the type of the family's housing?

☐ An apartment ☐ floor in a villa ☐ Small villa (less than 500 m^2)

☐ Medium size villa (500-1000 m^2) ☐ Large size villa (>1000 m^2)

13- How many of the children in the family are males?

☐ zero ☐ one ☐ two ☐ three ☐ four ☐ five or more

14- How many of the children in the family are females?

☐ zero ☐ one ☐ two ☐ three ☐ four ☐ five or more

15-How many of the children (both males and females) who are considered overweight or obese?

☐ zero ☐ one ☐ two ☐ three ☐ more than three (how many?): ……

16- Do you think that any of the parents (mother or father or both) are overweight or obese?

☐ none is obese ☐ only the mother ☐ only the father ☐ both parents

Part two:

17-During the school days (week days), how much time does your child spend watching television & videos games or uses iPad, computer or the internet (or all)?

☐ doesn't watch any ☐ an hour or less ☐ 2 hours ☐ 3 hours

☐ 4 hours ☐ 5 hours ☐ 6 hours ☐ > 6 hours (how much please): …..

18- During the weekend days, how much time does your child spend watching television & videos games or uses iPad, computer or the internet (or all)?

☐ doesn't watch any ☐ an hour or less ☐ 2 hours ☐ 3 hours

☐ 4 hours ☐ 5 hours ☐ 6 hours ☐ > 6 hours (how much please): …..

19- How long does your child usually sleep during the <u>school days (week days</u>)?

☐ 4 hours or less ☐ 5 hours ☐ 6 hours ☐ 7 hours

☐ 8 hours ☐ 9 hours ☐ 10 hours ☐ 11 hours or more

20- How long does your child usually sleep during the <u>weekend days</u>?

☐ 4 hours or less ☐ 5 hours ☐ 6 hours ☐ 7 hours

☐ 8 hours ☐ 9 hours ☐ 10 hours ☐ 11 hours or more

21- Does your child do any physical activity (or sports) that increase his/her heart rate or respiration substantially (other than his/her activity in school)?

☐ doesn't do any physical activity ☐ he/she is active for less than 30 min

☐ he/she is active for at least 30 min ☐ he/she is active for at least 60 min

☐ he/she is active for more than 60 min

22- Usually, how does your child go to school?

☐ walking ☐ using the family's car ☐ by school bus

<u>Part 3:</u>

23- During the school days, how many days does your child consume the breakfast meal at home before going to school?

☐ none ☐ once ☐ twice

☐ 3 times ☐ 4 times ☐ 5 times (daily)

24- During the weekend days, how many days does your child consume the breakfast meal at home (before 11 am)?

☐ none ☐ one day ☐ two days (both days)

25- In case the breakfast meal is prepared at home, who usually prepares it?

☐ the mother ☐ the father ☐ the maid (domestic helper) ☐ older sister/brother

☐ the child himself ☐ the meal is bought ready from a shop

☐ the meal is never prepared at home

26- In case your child <u>doesn't consume</u> the breakfast meal at home before going to school, what are the reasons for that (*you can choose more than one answers if applicable*)?

- ☐ the child wakes up late, so there is no time to consume breakfast
- ☐ the child doesn't feel hungry in the morning, so he/she doesn't eat breakfast at home
- ☐ the child is given a sandwich to consume on the way to school (please specify the type of food provided:……………………………………………………..…)
- ☐ the child is provided money to buy breakfast from the school canteen
- ☐ other reasons (please specify: …………..……………………………………..)

27- In case breakfast is consumed on a <u>regular basis at home</u>, what is usually the kind of meal prepared for your child (*you can choose more than one answers*)?

☐ boiled egg sandwich	☐ fried egg sandwich	☐ jam sandwich
☐ tuna sandwich	☐ peanut butter sandwich	☐ labneh sandwich
☐ thyme sandwich	☐ croissant	☐ nutella sandwich (chocolate & hazel nuts)
☐ hot dog	☐ Mortadella	☐ pizza
☐ spread cheese sandwich	☐ solid cheese sandwich	☐ French fries
☐ baked potatoes	☐ hamburger	☐ fava beans
☐ falafel	☐ chickpeas	☐ pancake
☐ cake or cookies	☐ donuts	☐ Oreo biscuits
☐ yogurt with fruits	☐ cheese pie	☐ thyme pie
☐ labneh pie		

- ☐ breakfast cereal (corn or oat grains), please specify the kind: …………….
- ☐ if none of the above, please specify the kind of breakfast consumed: ……………….

28- In case your child consumes breakfast cereals (corn or oat grains), what does he/she prefers?

☐ cereal with added sugar ☐ cereal without added sugar

29- In case your child consumes breakfast cereals, what does he prefers?

☐ cereal with fruits ☐ cereal without fruits

30- What are the fresh fruits that your child usually consumes as part of the breakfast meal (*you can choose more than one answers if applicable*)?

☐ doesn't consume any fruits with breakfast ☐ apple ☐ orange

☐ banana ☐ pear ☐ grapes ☐ other (please specify: ……….)

31- What does your child usually drink with breakfast (*you can choose more than one answers if applicable*)?

☐ doesn't drink anything ☐ water ☐ whole fat milk ☐ low fat milk

☐ fruit juice ☐ fruit drink ☐ tea ☐ tea with milk

☐ soft drinks ☐ other (specify): ……………..……

32- Is your child allergic to lactose (lactose intolerant)?

☐ Yes ☐ No

33- In case your child consumes breakfast at home during the school days, what time does he/she have it?

☐ before 6 am ☐ 6-6:30 am ☐ 6:30-7 am

34- In your opinion, what are the factors that influence your child most when choosing the type of food for breakfast consumption (*you can choose more than one answers if applicable*)?

☐ television advertisements ☐ friends ☐ parents

☐ siblings ☐ child's mood ☐ foods' taste

☐ other factors (please specify): ………………………………..……

35- Are you satisfied with the quality of the breakfast that your child consumes at home?

☐ Yes, satisfied ☐ somewhat satisfied ☐ Not satisfied

36- Does your child consume any foods or drinks from the school canteen (shop)?

☐ No ☐ Yes, consumes both foods and drinks

☐ consumes some foods only ☐ consumes some drinks only

37- Are you satisfied with the quality of the food that your child consumes from the school canteen (shop)?

☐ Yes, satisfied ☐ somewhat satisfied

☐ NO ☐ We have no information about the food in the school shop

38- Are you satisfied with the quality of the drinks that your child consumes from the school canteen (shop)?

☐ Yes, satisfied ☐ somewhat satisfied

☐ NO ☐ We have no information about drinks in the school shop

39- In case your child consumes foods or drinks from the school canteen (shop), how much money in Riyals do you give him/her for that?

☐ 2 or less ☐ 3-5 ☐ 6-10 ☐ more than 10

40- As a parent, how important for you is your child's breakfast?

☐ Very important ☐ somewhat important ☐ Not important

41- In your opinion, which meal you consider as most important for your child?

☐ Breakfast ☐ Lunch ☐ Dinner

42- In your opinion, does consuming breakfast regularly enhance the cognitive and school performance of your child?

☐ Yes ☐ No ☐ I don't know

43- Is having enough protein in the breakfast meal considered?

☐ Important ☐ Not important ☐ I don't know

44- Which of the following foods in the next table do you consider healthy or unhealthy? Please tick one choice for each item.

Type of food	Healthy	Somewhat healthy	Unhealthy
Solid cheese Sandwich			
Spread cheese sandwich			
Egg Sandwich			
Tuna Sandwich			
Thyme sandwich			
Peanut butter Sandwich			
Nutella Sandwich (chocolate and hazel nuts)			
Hot dog			
Mortadella			
Croissant			
French Fries			
Pizza			
Hamburger			
Fava Beans			
Chickpeas			
Pancake			
Donuts			
Yogurt with fruits			
Oreo Biscuits			
breakfast cereal (from whole weight or oat grains)			

Thank you for filling the questionnaire with care. Kindly send it back when completed to school with your child.
